# Effect of oral care gel on the quality of life for oral lichen planus in patients with chronic HCV infection

**DOI:** 10.1186/1743-422X-8-348

**Published:** 2011-07-12

**Authors:** Yumiko Nagao, Michio Sata

**Affiliations:** 1 Department of Digestive Disease Information & Research, Kurume University School of Medicine, Kurume, Fukuoka, 830-0011, Japan; 2 Division of Gastroenterology, Department of Medicine, Kurume University School of Medicine, Kurume, Fukuoka, 830-0011, Japan

## Abstract

**Background:**

Oral lichen planus (OLP) decreases the quality of life because it can cause spontaneous pain during eating and tooth-brushing and an uncomfortable feeling in the mouth. In addition, OLP may be associated with HCV-related liver disease.

We investigated the visual analogue scale (VAS) and effects of oral care gel, REFRECARE-H^®^, on patients with OLP associated with HCV infection.

**Results:**

Nine OLP patients (mean age 67.9 ± 7.6 years) with HCV-related liver diseases were recruited and their VAS score determined along with a biochemical examination of the blood. Types of OLP included erosive (6 patients) and reticular (3). REFRECARE-H^®^, an oral care gel (therapeutic dentifrice) containing hinokitiol, was applied by each patient as a thin layer on the oral membrane, after each meal and at bedtime for 30 days. Application of REFRECARE-H^® ^improved the quality of life in all terms of dry mouth, breath odor, oral freshness, oral pain during rest, oral pain at a mealtimes, taste disorder, loss of appetite, sleep disorder, depressive mood and jitteriness. VAS scores of dry mouth, breath odor, oral freshness, and sleep disorder were significantly increased 30 days after application of REFRECARE-H^® ^(P = 0.01, P = 0.05, P = 0.03, P = 0.04). VAS scores of oral pain at a mealtimes and taste disorder were increased 30 days after application of REFRECARE-H^® ^(P = 0.06). There was an absence of side effects.

**Conclusions:**

REFRECARE-H^® ^improved the quality of life for OLP. It is necessary for the hepatologist to educate patients regarding oral hygiene, as well as provide treatment of liver disease.

## Background

There are 170 million chronic hepatitis C virus (HCV) carriers throughout the world, of whom an estimated two million are in Japan. HCV is the major cause of hepatocellular carcinoma (HCC) in Japan, with 70% of cases being HCV-related [1]. In addition to Japan, the number of patients with HCV-related HCC is increasing worldwide [1].

HCV is associated with a broad spectrum of clinical and biological extrahepatic manifestations [2]. Chronic HCV infection has been linked to lichen planus, particularly with the involvement of the oral cavity [3]. Lichen planus is common among HCV-infected patients in Japan [4]. According to our studies, about 20% of patients infected with HCV may develop lichen panus [4]. The erosive type of oral lichen planus (OLP), in particular, can cause spontaneous pain during eating and tooth-brushing or an uncomfortable feeling in the mouth.

There are several reports of the coexistence of HCV infection with Sjögren's syndrome [5,6]. According to our research, 54 of 531 (10.2%) HCV-infected patients had a salivary flow below the normal value [7]. Patients with Sjögren's syndrome have an increased risk of periodontitis and candidiasis [8].

On April 1, 2008, EN Otsuka Pharmaceutical Co. Ltd. (Iwate, Japan) released REFRECARE-H^®^. REFRECARE-H^® ^is an oral care gel (therapeutic dentifrice) containing hinokitiol which can remove stains on the teeth and general oral debris, and is effective in the prevention of breath odor and gum diseases (periodontal infection and gingival inflammation). Hinokitiol is a natural chemical compound found in the wood of trees of the family Cupressaceae. Hinokitiol has significant antimicrobial efficacy against Staphylococcus aureus, Propionibacterium acnes, coronavirus, Trichophyton, and Candida albicans [9-11].

There are few reports showing that the lesions or symptoms of OLP decrease the quality of life for OLP patients with HCV-related liver disease [12]. The visual analogue scale (VAS) is a simple and frequently used method for evaluating variations in pain intensity [13].

In this study, we investigated the VAS and effects of REFRECARE-H^® ^on patients with OLP associated with HCV infection.

## Material and methods

### Subjects

This study included nine Japanese patients (4 males and 5 females) with OLP who were positive for HCV antibody (anti-HCV) and who visited our clinic at the Kurume University Hospital in Japan from November 2, 2011 to November 16, 2011. The patients ranged in age from 55 to 76 years, with an average age of 67.9 ± 7.6 years.

The biopsy specimens from all subjects showed histologic features characteristic of OLP. The types of OLP included erosive (6 patients) and reticular (3). All patients had been treated with topical administration of steroids and by elimination of irritating factors, for example, bad fillings and ill-fitting dentures.

### Serological assays

Sera from all nine patients were evaluated for white blood cell counts (WBC), red blood cell counts (RBC), hemoglobin (Hb) and platelets (PLT) and the following liver function tests were carried out: aspartate aminotransferase (AST), alanine aminotransferase (ALT), gamma-glutamyl transpeptidase (γ-GTP), alkaline phosphatase (ALP), lactate dehydrogenase (LDH), albumin (Alb), total bilirubin (T.Bil), and creatinine. The blood levels of hemoglobin A1c (HbA1c) and fasting blood glucose (FBS) were measured. Ultrasonographic examination was performed for all patients in order to investigate the shape of the liver and lesions occupying the liver. Computed tomography and liver biopsy were performed in some patients.

### Design of the administration of REFRECARE-H^®^

REFRECARE-H^®^, an oral care gel (therapeutic dentifrice) including hinokitiol, was applied by each patient as a thin layer on the oral membrane, after each meal and at bedtime for 30 days.

Informed consent according to Helsinki Declaration II for participation in the study was obtained from each patient.

### Salivary flow

We used a simple and low-cost test for detection of xerostomia and this required chewing on a piece of gauze for 2 min. A salivary flow rate ≤ 2 g/2 min was judged as decreased salivary secretion.

### Evaluation of VAS

**Figure 1:**
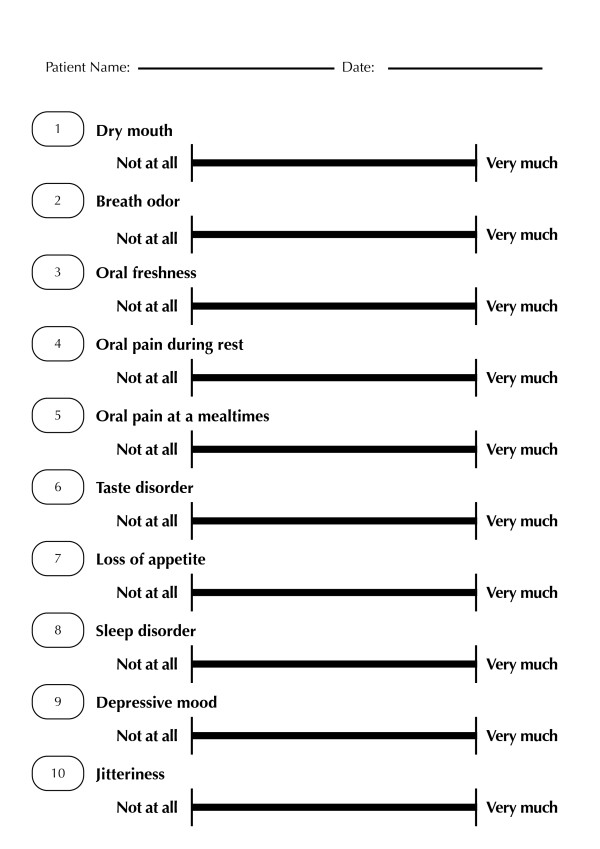
**VAS of 10 items**. A VAS is a horizontal line, 100 millimeters in length, anchored by word descriptors at each end.

A VAS is a horizontal line, 100 millimeters in length, anchored by word descriptors at each end, as illustrated in Figure 1. The patients marked on the line the point that they felt represented their perception of their current state, such as dry mouth, breath odor, oral freshness, oral pain during rest, oral pain at a mealtimes, taste disorder, loss of appetite, sleep disorder, depressive mood and jitteriness. The VAS score was determined by measuring in millimeters from the left hand end of the line to the point that the patient had marked.

### Statistical analysis

All data are expressed as mean ± standard error. Statistical comparisons before application of the REFRECARE-H^® ^and after 30 days were made using Wilcoxon's test. All statistical analyses were carried out using JMP Version 6 (SAS Institute, Cary, NC, USA). The level of statistical significance was defined as 0.05.

## Results

The characteristics of the nine patients studied are shown in Table 1. The diagnosis of liver disease included: chronic hepatitis C (n = 7), liver cirrhosis and post-treatment of HCC (n = 1), and post interferon (IFN) treatment for chronic hepatitis C (n = 1). Concomitant medical complications included: hypertension (n = 5), diabetes mellitus (DM)(n = 1) and asthma (n = 1).

**Table 1:** Characteristics of nine OLP patients with HCV-related liver diseases

**N0**.	Age	Sex	Oral disease	Liver disease	Concomitant medical complications
1	73	M	OLP of bilateral buccal mucosa and lower lip	CH-C	Hypertension

2	55	M	OLP of bilateral buccal mucosa, lower lip, and tongue	CH-C	DM

3	56	F	OLP of bilateral buccal mucosa and tongue	CH-C	

4	73	F	OLP of bilateral buccal mucosa and tongue	CH-C	Asthma

5	69	F	OLP of bilateral palatal plate	CH-C	Hypertension

6	74	M	OLP of tongue	CH-C post IFN SVR	Hypertension
					
			Post right tongue carcinoma		

7	76	F	OLP of bilateral buccal mucosa	LC-C	Hypertension
				
			Sjögren's syndrome	post-treatment of HCC	

8	67	F	OLP of right buccal mucosa	CH-C	

9	68	M	OLP of lower lip	CH-C	Hypertension

OLP: oral lichen planus

CH-C: chronic hepatitis type C

LC-C: liver cirrhosis type C

HCC: hepatocellular carcinoma

DM: diabetes mellitus

IFN: interferon

SVR: sustained virologic response

The distributions of VAS score before and after application of REFRECARE-H^® ^are as shown in Table 2. Each VAS score was lower after application of REFRECARE-H^® ^than before its use. Therefore, application of REFRECARE-H^® ^improved the quality of life according to all criteria. Subjective evaluations of dry mouth, breath odor, oral freshness and sleep disorders significantly improved 30 days after the application of REFRECARE-H^® ^(P = 0.01, P = 0.05, P = 0.03, P = 0.04, Table 2). Subjective evaluations of oral pain at a mealtimes and taste disorder improved 30 days after the application of REFRECARE-H^® ^(P = 0.06, Table 2).

**Table 2:** Effects of VAS score and laboratory data using REFRECARE-H^®^

		Before	After	P value
		mean ± SD	mean ± SD	
VAS score (mm)	dry mouth	56.22 ± 20.73	27.67 ± 19.29	**0.01**
	
	breath odor	39.33 ± 32.22	18.78 ± 26.06	**0.05**
	
	oral freshness	46.11 ± 25.03	21.44 ± 24.66	**0.03**
	
	oral pain during rest	34.00 ± 34.75	16.11 ± 24.62	0.09
	
	oral pain at a mealtimes	32.78 ± 38.33	19.00 ± 29.80	0.06
	
	taste disorder	34.56 ± 36.02	14.67 ± 26.48	0.06
	
	loss of appetite	25.56 ± 24.00	25.33 ± 25.73	0.89
	
	sleep disorder	50.56 ± 28.91	27.00 ± 34.01	**0.04**
	
	depressive mood	36.78 ± 30.86	27.00 ± 30.08	0.48
	
	jitteriness	42.22 ± 29.09	32.11 ± 29.80	0.19

Salivary flow (g/2 min)		4.88 ± 2.94	4.90 ± 2.05	0.82

Laboratory data	AST (U/L)	41.22 ± 24.37	38.78 ± 19.15	0.98
	
	ALT (U/L)	33.56 ± 23.31	33.44 ± 20.53	0.95
	
	γGTP (U/L)	24.89 ± 11.14	25.67 ± 11.78	0.39
	
	ALP (U/L)	293.44 ± 103.96	301.00 ± 100.99	0.95
	
	LDH (U/L)	186.50 ± 51.84	189.29 ± 59.08	0.31
	
	Alb (g/dL)	4.03 ± 0.46	3.96 ± 0.49	0.50
	
	T.Bil (mg/dL)	0.80 ± 0.40	0.84 ± 0.31	0.52
	
	Creatinine (mg/mL)	0.68 ± 0.14	0.67 ± 0.12	0.72
	
	FBS (mg/dL)	114.38 ± 19.81	103.13 ± 18.10	0.11
	
	HbA1c (%)	5.44 ± 0.55	5.54 ± 0.61	0.63
	
	AFP (ng/mL)	9.07 ± 11.34	8.41 ± 9.63	0.12
	
	WBC (/μL)	4411.11 ± 1095.95	4733.33 ± 1013.66	0.12
	
	RBC (/μL)	432.33 ± 37.46	440.11 ± 32.130	0.43
	
	Plt (/μL)	14.61 ± 4.88	15.26 ± 4.08	0.34
	
	Hb (g/dL)	13.23 ± 1.62	13.57 ± 1.69	0.14

We analyzed any changes in laboratory data before and after the application of REFRECARE-H^® ^(Table 2). There were no changes.

## Discussion

OLP is characterized by chronic inflammation and is often associated with severe pain and a burning sensation in the mouth. In particular, chronic erosive OLP is a painful disease inducing severe disability with weight loss and poor quality of life. López-Jornet reported that the quality of life in Spanish patients with OLP is reduced and that patient-centered measures should be considered in the management of OLP [12].

Oral Candida albicans also may be isolated from patients with OLP. Some Candida albicans isolates with special virulence attributes might be co-factors which contribute to the development of OLP, especially erosive OLP [14]. REFRECARE-H^®^, an oral care gel with efficacy against Candida albicans, improved the quality of life for OLP patients. Therefore, it is a useful agent for improvement of subjective complaints.

We previously reported that sensitivity to tastes and zinc levels are decreased in patients with HCV-associated liver disease [15]. Some patients had decreased sensitivity of taste despite the fact that they were unaware of their taste disorder. In addition, poor oral health has been reported for HCV-infected patients [16-19]. In our previous study, dental problems delayed the initiation of IFN therapy for a maximum of 105 days [7]. HCV-infected patients treated with IFN therapy should be managed by intensive oral care because of lower resistance to infection during the therapy. With regard to the painful OLP, it is just conceivable that an oral cavity with HCV infection is likely to become less healthy than one without HCV infection. It is necessary for a hepatologist to educate patients regarding oral hygiene as well as provide treatment of liver disease, so that the quality of life of patients with HCV-related liver disease does not decrease. Just as important as the apparent effectiveness of care using REFRECARE-H^® ^is the lack of side effects.

## Conclusions

In conclusion, we showed that oral care gel improved the subjective symptoms of all patients with OLP. The results of this study indicate that the use of REFRECARE-H^® ^could be effective in reducing the subjective symptoms and quality of life of patients with OLP.

## Abbreviations

OLP: oral lichen planus; HCV: hepatitis C virus; CH-C: chronic hepatitis C; LC-C: liver cirrhosis type C; HCC: hepatocellular carcinoma; DM: diabetes mellitus; IFN: interferon; SVR: sustained virologic response.

## Competing interests

The authors declare that they have no competing interests.

## Authors' contributions

YN carried out most of the data collection and drafted the manuscript. MS contributed to data analysis. All authors read and approved the final manuscript.

**Source of support**: This study was supported in part by a Grant-in-Aid for Scientific Research (C) (No.22592354) from the Ministry of Education, Culture, Sports, Science and Technology of Japan.
